# Thigh pain after total hip arthroplasty

**DOI:** 10.1097/JS9.0000000000000544

**Published:** 2023-06-28

**Authors:** Xiao-qiang Wang, Xiao-qiang Ren, De-an Qin

**Affiliations:** a Department of Medical Imaging Center, Shanxi Provincial People’s Hospital; b Department of Medical Information Center, Shanxi Provincial People’s Hospital; c Department of Orthopedics, Shanxi Provincial People’s Hospital, Taiyuan, Shanxi, China

*Dear Editor*,

Chronic pain after total hip arthroplasty (THA) may be problematic for many patients and usually presents a diagnostic and therapeutic challenge, especially for the well-fixed and well-positioned components. The etiologies of painful THA range from intrinsic to extrinsic, and a small patient group without an established etiology. Intrinsic etiologies include prosthetic infection, loosening, impingement, wear, misplacement, modulus mismatch, stress fracture, osteolysis, occult instability, etc. Extrinsic etiologies include myofascial/soft tissue pain, peripheral neuropathic or vascular pain, complex regional pain syndrome, metabolic disease, malignancy or metastases, referred pain, etc. There is so much opinion and so little science on the thigh pain after THA^1^. Locating the pain is essential and can suggest the underlying etiology. Typically, patients can localize the dull aching pain to a discrete area on the femur (i.e. anterolateral thigh) that correlates with the location of the prosthetic stem tip. Engh and Bobyn (1985) described three types of thigh pain: ‘start-up’ pain, end pain, and fatigue-fracture pain. Understanding the characteristics of chronic thigh pain after THA is of the utmost importance for detecting the potential etiologies. For example, pain presenting at the beginning of an activity that decreases when activity continues or improves with rest (‘start-up’ pain) should raise suspicion of prosthetic aseptic loosening, whereas pain not relieving with rest but continuing through the night is suggestive of infection. Comparison of prior serial radiographs can detect loosening (radiolucent lines>2 mm, stem subsidence>4 mm, or pedestal formation) and stem malalignment(variance>±5°)^2^. Technetium-99 methylene diphosphonate bone scintigraphy is a highly sensitive technique but has a very low specificity. Differentiation between infection and loosening is challenging, even when using a specific uptake pattern. It mainly depends on the radiotracer uptake extent and intensity. The scintigraphic pattern of infection is a diffuse and intense radiotracer uptake around the prosthesis. The scintigraphic pattern of loosening is a more focal uptake and more evident in the late images^3^.

All possible etiologies of thigh pain in patients with well-fixed, adequately aligned, and correctly sized uncemented THAs must be thoroughly evaluated and the most accepted etiology is inhomogeneous stress distribution between the calcar area and the femoral stem tip resulting from kinds of etiologies such as stem design, material composition, elasticity modulus, patient age, and bone quality. Unloading of the femoral calcar associated with increased stress at the femoral stem tip causes periprosthetic femoral remodeling such as calcar resorption (proximal stress shielding) and DFCH—fusiform enlargement of cortical bone at the femoral stem tip (distal stress concentration) (Fig. 1), which is attributed to the Wolff’s law^4^. Excess stress at the femoral stem tip can cause thigh pain or a subsequent possible periprosthetic stress fracture, though which is controversial^5^. More physiological material composition and stem designs have been modified to improve stress distribution and decrease the incidence of femoral remodeling and thigh pain.

**Figure 1:**
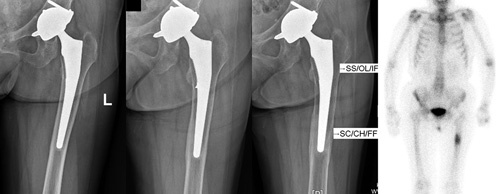
Serial radiographs of an 80-year-old woman with a 2-year moderate left anterior mid-thigh ‘start-up’ pain after uncemented total hip arthroplasty 7 years before. (A) A 4-year postoperative anteroposterior film showed no real radiolucency or distal femoral cortical hypertrophy (DFCH). (B) A 5-year postoperative film showed only a trace of DFCH. (C) Seven years after operation, there were extensive periprosthetic radiolucency in Gruen zones 1, 2, 6, and 7 and obvious DFCH in Gruen zones 3 and 5. (D) Technetium-99 methylene diphosphonate bone scintigraphy showed an increased uptake around the region of prosthesis stem tip. Inhomogeneous stress distribution [proximal stress shielding (SS) and distal stress concentration (SC)] after THA can cause periprosthetic femoral remodeling [proximal osteolysis (OL) and distal cortical hypertrophy(CH)] and subsequent possible periprosthetic stress fracture [proximal insufficiency fracture (IF) or distal fatigue fracture (FF)].

## Ethics approval and consent to participate

We have had the ethics approval and consent of the Shanxi Medical University ethics committee.

## Patient consent

We have had the patient’s written consent for publication.

## Sources of funding

There is no funding for the research.

## Author contribution

All authors had access to the data and a role in writing the manuscript. De-an Qin is the main study designer and supervision. The corresponding author has the right to grant on behalf of all authors.

## Conflicts of interest disclosure

We have read and understood the policy on declaration of interests and declare no competing financial interests.

## Research registration unique identifying number (UIN)

1. Name of the registry.
2. Unique Identifying number or registration ID.
3. Hyperlink to your specific registration (must be publicly accessible and will be checked): This is a letter to editor and the registry is not applicable.

## Guarantor

The Guarantor is De-an Qin.

## Acknowledgements

Not applicable.
